# Corrigendum to “The feasibility of testing whether *Fasciola hepatica* is associated with increased risk of verocytotoxin producing *Escherichia coli* O157 from an existing study protocol” [Prev. Vet. Med. 119 (2015) 97–104]

**DOI:** 10.1016/j.prevetmed.2015.06.004

**Published:** 2015-09-01

**Authors:** Graeme L. Hickey, Peter J. Diggle, Tom N. McNeilly, Sue C. Tongue, Margo E. Chase-Topping, Diana J.L. Williams

**Affiliations:** a Department of Epidemiology and Population Health, Institute of Infection and Global Heath, University of Liverpool, The Farr Institute@HeRC, Waterhouse Building, 1–5 Brownlow Street, Liverpool, L69 3GL, UK; b Moredun Research Institute, Pentlands, Science Park, Bush Loan, Penicuik, Midlothian, EH26 0PZ, UK; c SRUC (Scotland’s Rural College), West Mains Road, Edinburgh, EH9 3JG, UK; d Centre for Immunity, Infection and Evolution, Univer sity of Edinburgh, Ashworth Laboratories, Charlotte Auerbach Road, Edinburgh, EH9 3FL, UK; e Department of Infection Biology, Institute of Infection and Global Heath, University of Liverpool, Liverpool, L69 7ZJ, UK; f School of Veterinary Science, University of Liverpool, Leahurst Campus, Chester High Road, Neston, CH64 7TE, UK

The authors regret that there is an error in lines 617–622 of the R script (coinfection_updated.r; Supplemental Data). Namely, the list of parameters ‘pars0’ used in the calculation should have been ‘pars1’ in all instances. This resulted in a small error in the first sensitivity analysis in Section 4.2. Specifically, the third sentence should read:

“Power ranged from 13.5% (for OR = 1.2) to 99.5% (for OR = 3.0)”.

This error also affects Fig. 4; however the revised figure is visually indistinguishable from the original.

There is also a typographical error in the left hand side of Eq. (4). Specifically, it should read:

(4) $$E_{X|r_{j}=\hat{r}}\left[P\left[Y_{j}>0|\alpha_{j},\beta ,X_{1j},.....,X_{n_{j}j}\right]\right],$$

where the expectation is taken conditional on the average individual prevalence for *F. hepatica*, $\hat{r}$. The two instances of ‘$\alpha_{j}$ ’ in the integrand of the proceeding equation (that followed after averaging over the random effects and sample size distributions) should have been written as ‘*u*’. This does not affect the calculation of the random effects parameters. The authors would like to apologise for any inconvenience caused.

